# Supplementary material for: Reconciling Mining with the Conservation of Cave Biodiversity: A Quantitative Baseline to Help Establish Conservation Priorities
Source: PLoS One. 2016 Dec 20;11(12):e0168348. doi: 10.1371/journal.pone.0168348 (PMC5173368; doi:10.1371/journal.pone.0168348)
Supplement: S1 Dataset — (ZIP) [file pone.0168348.s002.zip › Taxa/Serra Norte/SN_2013/N4E/Lista N4E-65.pdf]

CAVIDADE N4E-0065

Classe	Ordem	Fam/Outros	Gên/Outros	Espécie	S-U	U-S	Zon.
Annelida	Oligochaeta			jovem	4	2	E
Arachnida	Acari	(Oribatida)		sp.3		X	E
Arachnida	Acari	(Parasitengonina)		sp.2		X	E
Arachnida	Acari	Ixodidae	*Amblyomma*	sp.1		X	E
Arachnida	Acari	Veigaiidae		sp.1		X	E
Arachnida	Amblypygi	Phrynidae	*Heterophrynus*	*longicornis*	1		E
Arachnida	Araneae	Anyphaenidae	aff. *Aljassa*	sp.1	X		E
Arachnida	Araneae	Araneidae	*Alpaida*	*antonio*	X		E
Arachnida	Araneae	Caponiidae		jovem	X		E
Arachnida	Araneae	Corinnidae		jovem	1		E
Arachnida	Araneae	Dipluridae		jovem	4		E
Arachnida	Araneae	Drymusidae	*Drymusa*	*spelunca*	X	X	E
Arachnida	Araneae	Nesticidae		jovem		X	E
Arachnida	Araneae	Ochyroceratidae		jovem		X	E
Arachnida	Araneae	Pholcidae	*Mesabolivar*	sp.1	X	X	E
Arachnida	Araneae	Salticidae		jovem	X		E
Arachnida	Araneae	Theridiidae	*Theridion*	sp.1	X		E
Arachnida	Araneae	Theridiosomatidae	*Plato*	sp.1	X	X	E
Arachnida	Opiliones	Sclerosomatidae	*Prionostema*	sp.1	2	3	E
Chilopoda	Geophilomorpha	Geophilidae		sp.1		1	E
Chilopoda	Scolopendromorpha	Cryptopidae	*Cryptops*	jovem		1	E
Chilopoda	Scolopendromorpha	Scolopocryptopidae	*Tidops*	sp.1		1	E
Chilopoda	Scutigeromorpha	Pselliodidae		jovem	3	1	E
Diplopoda	Polydesmida	Paradoxosomatidae		sp.1	X	1	E
Diplopoda	Spirostreptida	Pseudonannolenidae	*Pseudonannolene*	jovem	2		E
Entognatha	Collembola	Paronellidae		sp.1	X	X	E
Entognatha	Collembola	Paronellidae		sp.6	X		E
Entognatha	Diplura	Campodeidae		sp.1	X	X	E
Insecta	Coleoptera	Carabidae		sp.3		X	E
Insecta	Coleoptera	Chrysomelidae		sp.3	X		E
Insecta	Coleoptera	Staphylinidae	Pselaphinae	sp.34	X		E
Insecta	Coleoptera	Staphylinidae	Pselaphinae	sp.37	X		E
Insecta	Coleoptera	Staphylinidae		sp.1	X		E
Insecta	Coleoptera	Staphylinidae		sp.26	X		E
Insecta	Coleoptera	Staphylinidae		sp.53	X		E
Insecta	Diptera	Mycetophilidae		sp.	X		E
Insecta	Diptera	Psychodidae	Phlebotominae	sp.		X	E
Insecta	Diptera	Sciaridae		sp.	X		E
Insecta	Diptera	Simuliidae		sp.		X	E
Insecta	Diptera	Tipulidae	Tipulinae	sp.	X	X	E
Insecta	Heteroptera	Cydnidae		jovem	X		E
Insecta	Heteroptera	Lygaeidae		jovem		X	E
Insecta	Heteroptera	Reduviidae	Emesinae	sp.		1	E
Insecta	Homoptera	Cixiidae		sp.3		X	E
Insecta	Hymenoptera	Formicidae	*Camponotus*	sp.1	X		E
Insecta	Hymenoptera	Formicidae	*Camponotus*	sp.2		X	E
Insecta	Hymenoptera	Formicidae	*Crematogaster*	sp.1	X		E
Insecta	Hymenoptera	Formicidae	*Gnamptogenys*	sp.1	X	X	E
Insecta	Hymenoptera	Formicidae	*Hypoponera*	sp.1		X	E

Insecta	Hymenoptera	Formicidae	*Pachycondyla*	sp.1	X	X	E
Insecta	Hymenoptera	Formicidae	*Paratrechina*	sp.2	X		E
Insecta	Hymenoptera	Formicidae	*Pheidole*	sp.1		X	E
Insecta	Hymenoptera	Formicidae	*Solenopsis*	sp.1	X		E
Insecta	Isoptera	Termitidae	*Nasutitermes*	sp.	X	X	E
Insecta	Lepidoptera			jovem		X	E
Insecta	Orthoptera	Gryllidae	Nemobiinae	jovem		1	E
Insecta	Orthoptera	Phalangopsidae	*Paraclodes*	jovem	9	7	E
Insecta	Thysanura	Ateluridae		sp.2		X	E
Malacostraca	Isopoda	Dubioniscidae		sp.1		X	E
Malacostraca	Isopoda	Philosciidae		sp.1	X	X	E
Symphyla		Scutigerellidae		sp.		X	E
Amphibia	Anura	Strabomantidae	*Pristimantis*	cf. *fenestratus*	5		E
Mammalia	Chiroptera	Emballonuridae	*Peropteryx*	*kappleri*	3		E
